# Case Report: Tumor lysis syndrome in advanced, massive hepatocellular carcinoma with main portal vein invasion following atezolizumab plus bevacizumab therapy

**DOI:** 10.3389/fonc.2025.1624908

**Published:** 2025-09-03

**Authors:** Tien-Shin Chou, Chun-Feng Wu, Chih-Lang Lin, Chao-Wei Hsu

**Affiliations:** ^1^ Division of Gastroenterology, Department of Internal Medicine, Keelung Chang Gung Memorial Hospital, Keelung, Taiwan; ^2^ Division of Oncology, Department of Internal Medicine, Keelung Chang Gung Memorial Hospital, Keelung, Taiwan; ^3^ Department of Gastroenterology and Hepatology, Linkou Chang Gung Memorial Hospital and Chang Gung University College of Medicine, Taoyuan, Taiwan

**Keywords:** case report, tumor lysis syndrome, hepatocellular carcinoma, atezolizumab plus bevacizumab, systemic therapy, main portal vein invasion

## Abstract

**Background:**

Tumor lysis syndrome (TLS) is extraordinarily rare in solid tumors. However, the combination of atezolizumab and bevacizumab (AB) in advanced hepatocellular carcinoma (HCC) has raised concerns for abrupt metabolic derangements. We describe a fatal episode of TLS precipitated by AB in HCC with main portal vein (Vp4) invasion, highlighting the need for vigilant risk stratification and early biochemical surveillance for high-tumor-burden disease.

**Case presentation:**

A 65-year-old man with chronic hepatitis B and massive HCC (Vp4 invasion) had relatively preserved organ function at baseline. Two days after initiating atezolizumab (1,200 mg) and bevacizumab (15 mg/kg), he developed fever (38.5°C) and tachycardia (heart rate 112 beats/min [bpm]), rapidly progressing to circulatory shock on day 3. Laboratory workup revealed hyperuricemia (12.4 mg/dL), hyperphosphatemia (12.9 mg/dL), hyperkalemia (6.8 mmol/L), hypocalcemia (7.7 mg/dL), and acute kidney injury, meeting the Cairo–Bishop criteria for TLS. Imaging demonstrated abrupt cystic changes of the hepatic mass, suggesting rapid tumor necrosis. Despite aggressive fluid resuscitation and the initiation of hemodialysis, the patient succumbed to multiorgan failure within eight days after initiating AB.

**Conclusion:**

This case underscores that advanced HCC with extensive vascular invasion is at risk of severe TLS shortly after potent immuno-antiangiogenic therapy. Clinicians should incorporate thorough baseline risk assessments, prompt laboratory monitoring, and timely intervention into standard care algorithms for high-risk HCC. The rapid metabolic collapse observed here serves as a reminder that while combination therapy holds promise for improving survival in advanced HCC, it can also lead to life-threatening complications in specific subgroups. Careful selection of therapeutic options and shared decision-making with patients are critical to balancing potential benefits against severe adverse events, such as TLS.

## Introduction

Hepatocellular carcinoma (HCC) is the most common primary liver cancer, often linked to chronic liver disease caused by viral hepatitis and alcohol (1). Immune checkpoint inhibitors (ICIs) and anti-angiogenic agents have revolutionized systemic therapy for advanced HCC, with the combination of atezolizumab and bevacizumab (AB) demonstrating significant survival benefits (2). While effective, these therapies carry risks, including tumor lysis syndrome (TLS), a rare but potentially fatal complication in solid tumors (3). Although rare, the increasing use of potent systemic treatments, particularly in patients with high tumor burden or Vp4 invasion, necessitates heightened awareness of TLS. Here, we present a case of fatal TLS following AB in advanced HCC, underscoring the need for risk assessment and preventive measures.

## Case presentation

**Figure 1:**
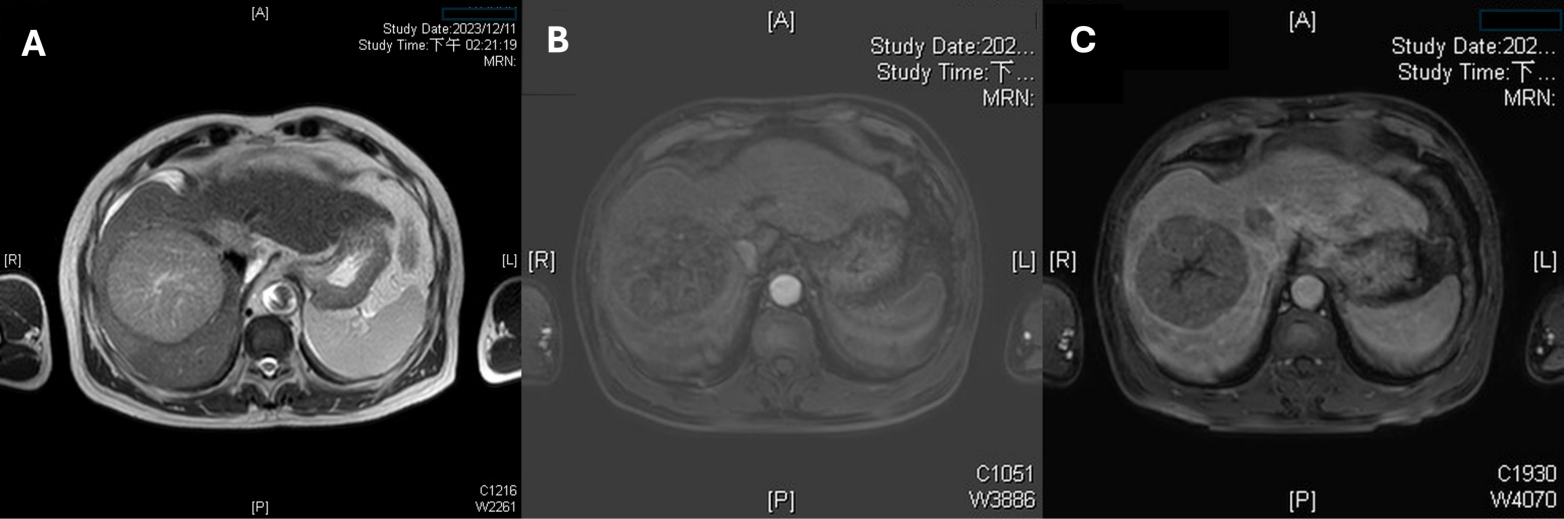
Multiphase liver magnetic resonance imaging (MRI) demonstrating the index tumor. **(A)** An axial T2-weighted image reveals a hepatocellular carcinoma in Couinaud segment 8 with marked hyperintensity and central necrosis. **(B)** An axial arterial-phase MRI sequence shows intense, heterogeneous hyperenhancement of the 8.5-cm lesion. **(C)** A portal venous–phase (60 s) image demonstrates washout with a peripheral capsule-like rim, meeting Liver Imaging Reporting and Data System (LI-RADS) category 5 diagnostic criteria.

**Figure 2:**
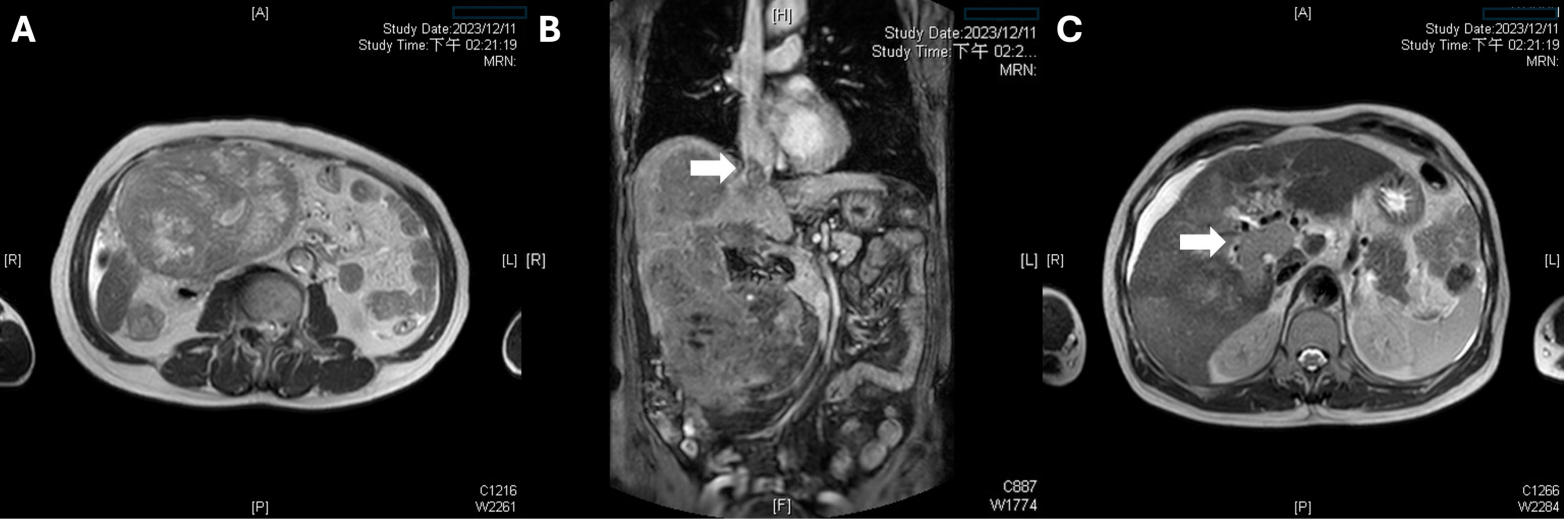
Multifocal disease and macrovascular invasion by tumor thrombus. **(A)** A second 14-cm hepatocellular carcinoma in Couinaud segment 6 exhibits high T2 signal intensity with internal septations, confirming multifocal disease. **(B)** A coronal MRI reveals a continuous tumor thrombus extending from the right hepatic vein into the suprahepatic inferior vena cava. **(C)** An axial portal venous–phase image demonstrates tumor thrombus filling the right and main portal veins.

A 65-year-old male with a history of chronic hepatitis B, who was not under regular medical surveillance, presented to our emergency department with abdominal pain. Liver magnetic resonance imaging (MRI) demonstrated two hypervascular masses in Couinaud segments 6 (14 cm) and 8 (8.5 cm) (4). Both lesions showed arterial hyperenhancement with subsequent washout and a delayed enhancing capsule, meeting LI-RADS 5 diagnostic criteria (**Figures 1**, **2A**) (5). A coronal MRI (**Figure 2B**) shows a continuous tumor thrombus extending from the right hepatic vein into the suprahepatic inferior vena cava (IVC). An axial portal venous–phase image (**Figure 2C**) further demonstrates tumor thrombus filling the right and main portal veins, corresponding to Vp4 involvement and Barcelona Clinic Liver Cancer (BCLC) stage C (Vp4 + IVC invasion) (6). The disease was staged as T4N0M0 according to the American Joint Committee on Cancer (AJCC) system (7). Despite advanced disease, the patient maintained an Eastern Cooperative Oncology Group (ECOG) performance status of 1 (8).

Baseline laboratory studies revealed an alpha-fetoprotein (AFP) level of 1.7 ng/mL and an elevated protein induced by vitamin K absence or antagonist-II (PIVKA-II) level of 8539 ng/mL. Renal and liver function were stable (creatinine 0.79 mg/dL, total bilirubin 0.5 mg/dL, albumin 3.7 g/dL), and there were mildly elevated liver enzymes (aspartate aminotransferase [AST] 78 IU/L, alanine aminotransferase [ALT] 65 IU/L). The platelet count was 98 × 10^9^/L, and white blood cell count 5.8 × 10^9^/L. Prothrombin time (INR 1.3) was mildly prolonged, likely secondary to underlying liver disease. Esophagogastroduodenoscopy revealed no esophageal or gastric varices. Based on these findings, the tumor board recommended systemic therapy with AB.

**Figure 3:**
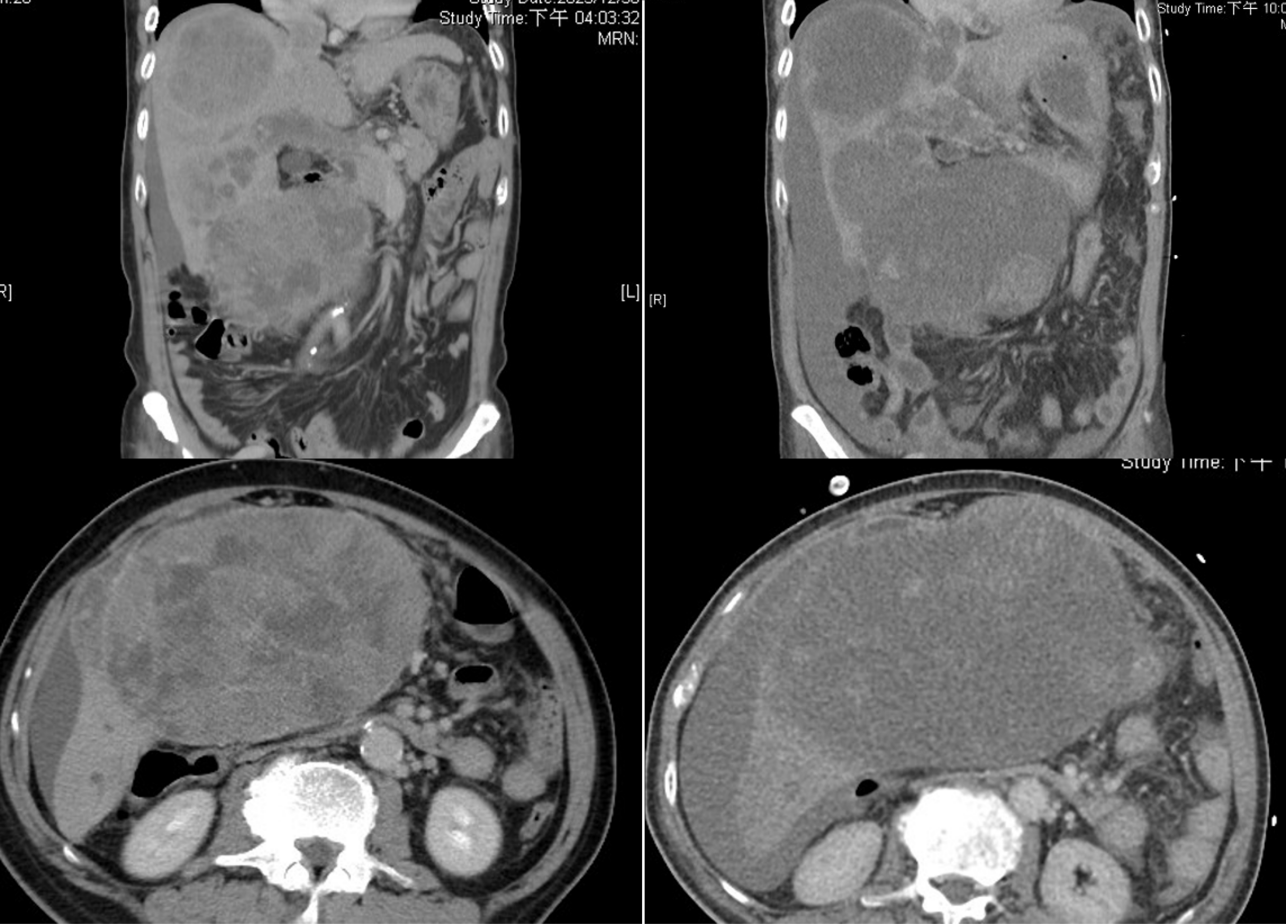
Computed tomography (CT) images. Left panel (pre-treatment): A contrast-enhanced CT scan demonstrates large masses involving Couinaud segments V and VIII, as well as evidence of main portal vein thrombosis. Right panel (post-treatment): A contrast-enhanced CT scan reveals cystic changes within the hepatic mass and no active contrast extravasation, findings suggestive of rapid tumor regression.

**Figure 4:**
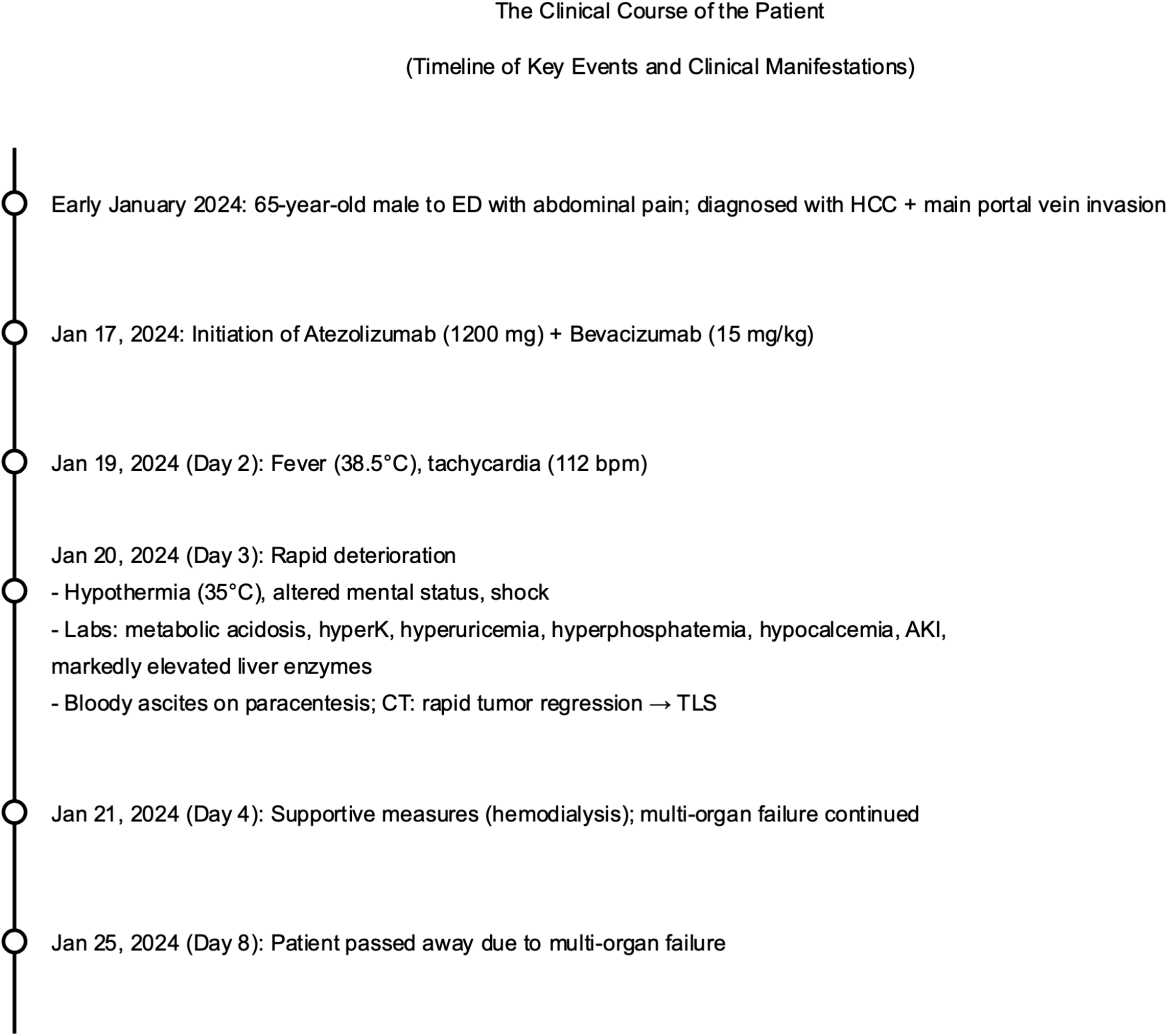
The clinical course of the patient. ED, emergency department; HCC, hepatocellular carcinoma; TLS, tumor lysis syndrome; AKI, acute kidney injury; CT, computed tomography; bpm, beats per minute; mg, milligram; mg/kg, milligram per kilogram; °C, degrees Celsius.

On January 17, 2024, the patient initiated combination therapy with atezolizumab (1,200 mg) and bevacizumab (15 mg/kg). On day 2, the patient developed fever (38.5°C) and tachycardia (112 beats/minute). He received supportive care and remained under close observation. On day 3, his condition rapidly deteriorated with hypothermia (35°C), altered mental status, and cold extremities, indicative of shock. Laboratory tests revealed severe metabolic acidosis (pH 7.06; bicarbonate 3.6 mmol/L), acute kidney injury (creatinine 2.84 mg/dL), hyperkalemia (potassium 6.8 mmol/L), hypocalcemia (calcium 7.7 mg/dL), hyperphosphatemia (phosphate 12.9 mg/dL), and hyperuricemia (uric acid 12.4 mg/dL), meeting the Cairo–Bishop criteria for TLS (9). Liver enzymes were markedly elevated (AST 8336 IU/L, ALT 1556 IU/L), and total bilirubin increased to 7.4 mg/dL and the INR increased to 2.0. Hemoglobin declined from 9.5 g/dL at admission to 7.5 g/dL, prompting transfusion of two units of packed red cells. Abdominal paracentesis yielded bloody ascites. Emergent computed tomography angiography (**Figure 3**) demonstrated the pretreatment tumor on the left, whereas the post-treatment image on the right revealed cystic changes without active contrast extravasation and with only minimal hemoperitoneum, findings consistent with rapid tumor regression rather than tumor hemorrhage. Nevertheless, hemoglobin remained 7.3 g/dL the following day. Subsequently, two additional units of packed red cells were transfused, and two days later hemoglobin increased to 8.4 g/dL after a cumulative total of four units of packed red cells had been administered. Despite supportive measures, including hemodialysis for hyperkalemia and acidosis, the patient developed multiorgan failure and died eight days after treatment initiation. The clinical course was consistent with TLS triggered by the combination therapy (**Figure 4**).

## Discussion

From a clinical perspective, patients with advanced HCC who carry a heavy tumor burden and vascular invasion can face TLS after only a brief course of systemic therapy, a risk highlighted by the present case. In this patient, the rapid tumor cell death triggered by AB led to a sudden cascade of metabolic derangements. To our knowledge, the case we present is the first report of TLS following HCC treatment with AB. Initially, the patient’s rapid clinical deterioration raised concern for immune-related adverse events (irAEs), which can occur with ICIs such as atezolizumab. However, irAEs usually develop weeks to months after initiation of ICIs (10), whereas this patient’s symptoms emerged within two days. The clinical course, together with laboratory and imaging findings, ultimately pointed to TLS, a very uncommon complication in this setting (3, 11).

TLS is well recognized in hematologic malignancies, whereas its occurrence in solid tumors remains sporadic (12). Although the overall incidence of TLS in HCC remains low relative to the global HCC burden, TLS in HCC has reported mortality rates of 50%–70% in small case series, a figure comparable to that observed in hematologic malignancies (12, 13). This high mortality may be partly related to delayed recognition and underappreciation of TLS in solid tumors (14). Reports show that TLS in HCC can occur across different treatment modalities, including chemotherapy, locoregional therapies, targeted therapies, ICIs, and even spontaneously (12). Transarterial chemoembolization and sorafenib together accounted for nearly 60% of reported TLS episodes in HCC, with lenvatinib, hepatic arterial infusion chemotherapy (HAIC) and spontaneous cases comprising most of the remainder. TLS typically occurs rapidly, with a median onset of 2 days after TACE and 7 days after sorafenib (12). In the only published case of nivolumab combined with sorafenib, TLS was diagnosed on day 9 after sorafenib initiation (15). Fatal events have occurred across treatment modalities, including sorafenib, TACE, and ICIs; nevertheless, every fatal case exhibited bulky, multifocal disease with Vp3/4 portal vein invasion or a serum α-fetoprotein level above 10,000 ng/mL, indicating that tumor burden, rather than any single drug class, is the predominant risk factor. These findings support applying the same risk-stratified prophylaxis, consisting of aggressive hydration with or without rasburicase, together with metabolic monitoring every 6 to 8 hours that is recommended for TACE and tyrosine kinase inhibitors whenever high-response regimens, including ICIs, are initiated in similarly high-risk patients. Furthermore, the occurrence of fatal TLS following AB in the present case emphasizes critical safety concerns associated with ICIs in advanced HCC, particularly in patients with high-risk features such as Vp4 portal vein invasion. This rare yet severe complication prompts a reassessment of the risk–benefit profile of AB in Vp4 portal vein invasion HCC and careful reevaluation of therapeutic approaches for this challenging subgroup.

According to the BCLC staging system, HCC with portal vein invasion has an expected median survival of approximately two years (6). However, patients whose tumors invade the Vp4 portal vein have a poor prognosis, characterized by a low objective response rate and a median overall survival (OS) often only a few months (16). Current guidelines recommend AB as a first-line systemic therapy for advanced HCC; however, its benefit appears attenuated in patients with high-risk features such as Vp4 portal vein invasion or a tumor burden ≥50% (17, 18). A pooled analysis of randomized controlled trials (RCTs) showed no statistically significant advantage of AB over sorafenib in this subgroup with respect to overall survival (OS; 7.6 vs. 5.5 months; HR, 0.62; 95% CI, 0.39–1.00), progression-free survival (PFS; 5.4 vs. 2.8 months; HR, 0.74; 95% CI, 0.47–1.17), or objective response rate (ORR; 25% vs. 14%; OR, 2.04; 95% CI, 0.72–6.77) (19). In addition to its limited efficacy, AB carries a markedly higher incidence of esophageal variceal hemorrhage in HCC with Vp4 invasion (14% vs. 0% with sorafenib) (19). Patients with variceal bleeding face a high risk of decompensation or death and will typically require deferral or discontinuation of further immunotherapy. Moreover, up to 25% of patients with HCC and Vp4 invasion may experience serious AEs, such as irAEs (20). These observations raise important questions about the suitability of AB in high-risk HCC. Alternative therapies with potentially fewer AEs and better quality-of-life outcomes should be carefully considered and discussed with patients through shared decision-making.

According to current guidelines, several other first-line treatment options exist for patients with advanced HCC, such as lenvatinib or the durvalumab/tremelimumab combination (18). However, evidence supporting their use in patients with Vp4 invasion remains limited, as these patients were specifically excluded from the pivotal REFLECT and HIMALAYA trials (21, 22). Consequently, their benefit in HCC with Vp4 invasion remains uncertain, and only real-world data suggest that lenvatinib may outperform sorafenib in this setting (23). Additionally, a preliminary study indicated that lenvatinib exhibited comparable efficacy and safety to AB in HCC complicated by portal vein tumor thrombosis (24). Although the LEAP-002 study comparing lenvatinib plus pembrolizumab versus lenvatinib alone excluded patients with Vp4 invasion, subgroup analyses consistently favored combination therapy across all evaluated populations (25). Notably, the benefit trend was particularly pronounced in patients with high-risk features, including macrovascular invasion, implying that patients with Vp4 invasion may derive benefit from a tyrosine kinase inhibitor (TKI)-ICIs combination regimen, a hypothesis warranting further investigation (25).

Before the advent of ICIs, HAIC with fluorouracil, leucovorin, and oxaliplatin (HAIC-FO) already demonstrated significant superiority over sorafenib in the Phase III FOHAIC-1 trial, extending PFS (7.7 vs. 2.9 months; HR, 0.38, 95% CI 0.25–0.58) and OS (10.8 vs. 5.7 months; HR, 0.34, 95% CI 0.22–0.54) (26). Consistently, a real-world propensity-score-matched study found no difference in OS or PFS between AB and HAIC, whereas HAIC achieved a significantly higher disease-control rate (DCR; 87.9% vs. 75.9%) (27). Furthermore, combining HAIC with radiotherapy might be effective for advanced HCC with Vp4 (28). A recent retrospective study also suggests that combining therapeutic approaches including TKIs, PD-1 inhibitors, and HAIC may yield synergistic effects and encouraging preliminary efficacy in advanced HCC patients exhibiting high-risk features (29). This approach yielded an ORR of 78.3%, a DCR of 92.8%, a median PFS of 9.8 months, and a median OS of 19 months (29). Importantly, only 9.5% of patients experienced serious adverse events, although prospective studies are required to confirm these findings.

In our case, combination therapy with AB resulted in a significant tumor response, as evidenced by cystic changes on imaging. However, this rapid response also contributed to the onset of TLS. The case underscores the importance of identifying individual patient risk factors, such as tumor size and vascular invasion, prior to initiating potent anti-cancer therapies. Rigorous early-phase monitoring, including frequent assessments of electrolytes, renal function, and tumor lysis markers (e.g., uric acid and lactate dehydrogenase), is warranted in patients with Vp4 portal vein invasion or a tumor burden ≥50%. Prophylactic measures, such as aggressive intravenous hydration and the use of agents like allopurinol or rasburicase, should be considered (30). Despite early recognition and aggressive treatment in our patient, TLS progressed to fatal multi-organ failure, illustrating the need for heightened vigilance and immediate intervention.

## Conclusion

Using the BCLC staging system alone to predict survival is suboptimal for the subgroup of HCC patients with Vp4 portal vein invasion, who generally have a poor prognosis and limited treatment response rates. While combination therapy with AB may offer survival benefits in advanced HCC, only HAIC has thus far been shown in a RCT to be significantly superior to sorafenib in patients with advanced HCC and Vp4 invasion. This case highlights the critical importance of proper patient selection and close monitoring. Beyond irAEs, clinicians should be aware of TLS in patients with extensive HCC and implement appropriate preventive strategies. In addition, incorporating a risk–benefit analysis based on RCT data and real-world evidence into shared decision-making, along with patients’ preferences and values, helps ensure the selection of the most appropriate treatment while preserving quality of life in this challenging subgroup.

## Data Availability

The raw data supporting the conclusions of this article will be made available by the authors, without undue reservation.
